# Differential Effects of Mesenchymal Stem Cell- and Natural Killer Cell-Derived Extracellular Vesicles on Cisplatin Responsiveness in Endometrial Cancer Cells

**DOI:** 10.3390/ijms27135842

**Published:** 2026-06-28

**Authors:** Ren-Jun Hsu, Cheng-Shuo Huang, Ming-Kung Yeh, Zheng-Zong Lai, Cheng-Ping Yu, Jar-Yi Ho, Fung-Wei Chang

**Affiliations:** 1 Cancer Center, Hualien Tzu Chi Hospital, Buddhist Tzu Chi Medical Foundation, Hualien 970473, Taiwan; hsurnai@gmail.com; 2 School of Medicine, Tzu Chi University, Hualien 970374, Taiwan; 3 Department of BioMedical Engineering, Ming Chuan University, Taoyuan 333321, Taiwan; 4 Graduate Institute of Life Sciences, College of Biomedical Sciences, National Defense Medical University, Taipei 114, Taiwan; qo4m4443151@gmail.com (C.-S.H.); mkyeh2004@precisionthera.com (M.-K.Y.); cpyupath@yahoo.com.tw (C.-P.Y.); jaryiho@gmail.com (J.-Y.H.); 5 Graduate Institute of Pathology and Parasitology, College of Medicine, National Defense Medical University, Taipei 114, Taiwan; 6 Department of Pathology, Tri-Service General Hospital, National Defense Medical University, Taipei 114, Taiwan; 7 School of Pharmacy, College of Pharmacy, National Defense Medical University, Taipei 114201, Taiwan; 8 Independent Researcher, Taipei 116053, Taiwan; laizengzong@gmail.com; 9 Department of Obstetrics and Gynecology, Tri-Service General Hospital, National Defense Medical University, Taipei 114201, Taiwan; 10 Department of Obstetrics and Gynecology Medicine, School of Medicine, College of Medicine, National Defense Medical University, Taipei 11490, Taiwan; 11 Department of Health Promotion and Health Education, National Taiwan Normal University, Taipei 10610, Taiwan; 12 Department of Obstetrics and Gynecology, Taipei City Hospital, Taipei 103212, Taiwan

**Keywords:** extracellular vesicles, endometrial cancer, cisplatin resistance, apoptosis, cell-cycle regulation

## Abstract

Cisplatin (cis-diamminedichloroplatinum(II) [DDP]) is a key chemotherapeutic agent for advanced endometrial cancer; however, chemoresistance substantially limits its clinical benefit. Extracellular vesicles (EVs) mediate intercellular communication and influence tumour cell behaviour and therapeutic response. We investigated whether mesenchymal stem cell-derived extracellular vesicles (MSC-EVs) and natural killer cell-derived extracellular vesicles (NK-EVs) modulate cisplatin responsiveness in endometrial cancer cells (RL95-2 and HEC-1A). MSC-EVs and NK-EVs were isolated and characterised using nanoparticle tracking analysis, scanning electron microscopy, and EV marker profiling. MSC-EVs and NK-EVs reduced RL95-2 and HEC-1A cell viability in a dose-dependent manner, with MSC-EVs exhibiting substantial effects at lower particle concentrations. In a cisplatin-resistant HEC-1A (HEC-1A DDP-R) model, MSC-EVs were associated with greater reductions in cell viability under cisplatin treatment conditions, whereas NK-EVs showed comparatively modest effects. Mechanistic analyses demonstrated altered expression of apoptosis- and cell cycle–related proteins, including increased cleaved poly(ADP-ribose) polymerase and cleaved caspase-3 levels and reduced cyclin A and cyclin D1 expression following MSC-EV treatment. Annexin V-fluorescein isothiocyanate/propidium iodide flow cytometry demonstrated increased apoptotic cell populations after MSC-EV treatment, with MSC-EV + DDP co-treatment resulting in the highest apoptotic fraction in chemoresistant HEC-1A cells. Collectively, these findings indicate that MSC-EVs are associated with altered cellular responses to cisplatin in chemoresistant endometrial cancer cells, accompanied by changes in apoptosis-related protein expression, apoptotic cell populations, and cell-cycle regulators. Further investigation is required to determine their mechanistic role and therapeutic potential in overcoming chemoresistance.

## 1. Introduction

Endometrial cancer is among the most common gynaecological malignancies worldwide, with a rising incidence associated with ageing populations and metabolic disorders [1]. Although early-stage endometrial cancer is often associated with favourable outcomes, patients with advanced, recurrent, or high-grade disease generally have a poorer prognosis and more limited therapeutic options [2,3,4]. Chemotherapy, particularly platinum-based regimens (e.g., cisplatin or carboplatin) [5,6], is a key component in the treatment of advanced endometrial cancer. However, the development of chemoresistance to platinum-based chemotherapy, particularly cisplatin, considerably limits therapeutic efficacy and poses a major clinical challenge [7,8].

Cisplatin resistance develops through multiple mechanisms, such as enhanced DNA damage repair, dysregulation of apoptotic signalling, alterations in cell-cycle control, and tumour microenvironment-mediated survival signalling [9,10]. These mechanisms enable tumour cells to evade drug-induced cytotoxic stress, leading to tumour persistence and disease progression [11,12]. Consequently, strategies that restore chemosensitivity in drug-resistant endometrial cancer cells are of considerable clinical interest [13].

Extracellular vesicles (EVs) are critical mediators of intercellular communication. EVs carry proteins, lipids, and nucleic acids reflective of their parental cells and can substantially influence recipient cell behaviour [14,15]. Increasing evidence suggests that EV-mediated signalling contributes to tumour progression, drug resistance, immune modulation and therapeutic response [16,17,18,19]. Importantly, EVs derived from different cellular sources exhibit distinct functional properties owing to differences in their molecular cargo and membrane composition. Accumulating data indicate that EVs might participate in the development and dissemination of chemoresistant phenotypes among tumour cells [20,21,22].

Mesenchymal stem cell-derived extracellular vesicles (MSC-EVs) have been extensively studied for their regenerative and immunomodulatory properties. In oncological contexts, MSC-EVs modulate tumour cell proliferation, survival pathways, and responses to chemotherapy, although their effects appear to be context-dependent [23,24,25]. In contrast, natural killer cell-derived extracellular vesicles (NK-EVs) are primarily recognised for their immune effector functions. NK-EVs carry cytotoxic molecules capable of influencing tumour cell viability [26,27,28]. However, the comparative roles of MSC-EVs and NK-EVs in modulating chemoresistance mechanisms in endometrial cancer remain insufficiently defined.

To address the clinical problem of cisplatin resistance in endometrial cancer, this study investigated whether EV-based interventions can enhance the efficacy of cisplatin. Specifically, we examined the effects of MSC-EVs and NK-EVs, alone and in combination with cisplatin, on cisplatin-resistant endometrial cancer cells and explored the underlying mechanisms, focusing on apoptosis and cell-cycle regulation. We hypothesised that the origin of MSC-EVs and NK-EVs determines their functional impact on chemoresistance. We also hypothesised that MSC-EVs, because of their distinct molecular cargo, would enhance cisplatin responsiveness in chemoresistant endometrial cancer cells by reactivating apoptotic pathways and suppressing cell-cycle progression, while comparatively, NK-EVs would exert limited modulation of intrinsic drug-response pathways.

## 2. Results

### 2.1. Characterisation of MSC-EVs and NK-EVs

MSC-EVs and NK-EVs were first characterised in accordance with established EV-related MISEV2023 guidelines [29].

**Figure 1:**
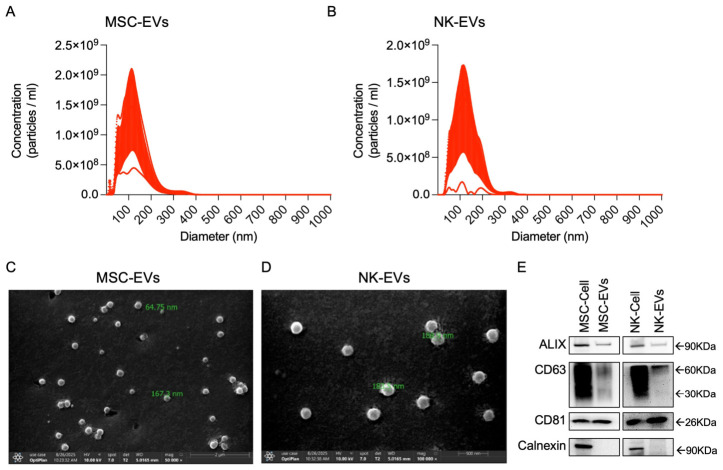
Characterisation of MSC-EVs and NK-EVs. (**A**,**B**) NTA showing the size distribution and particle concentration of MSC-EVs and NK-EVs. MSC-EVs exhibited a mean diameter of 95.5 nm and a mode size of 65.5 nm, while NK-EVs showed a mean diameter of 110.5 nm and a mode size of 85.5 nm. (**C**,**D**) SEM images demonstrating the morphology of MSC-EVs and NK-EVs. MSC-EVs and NK-EVs displayed round membrane-bound vesicles within the nanoscale range; Representative SEM images of MSC-EVs and NK-EVs are shown. Images were acquired at different magnifications and are presented to illustrate vesicular morphology. (**E**) Western blot analysis of EV marker proteins. MSC-EVs and NK-EVs were positive for the EV markers ALIX, CD63, and CD81, whereas the endoplasmic reticulum marker Calnexin was detected in parental cells but not in MSC-EVs and NK-EVs, indicating minimal cellular contamination. ALIX: apoptosis-linked gene 2-interacting protein X; CD: cluster of differentiation; MSC-EV: mesenchymal stem cell-derived extracellular vesicle; NK-EV: natural killer cell-derived extracellular vesicle; NTA: nanoparticle tracking analysis; SEM: scanning electron microscopy.

In nanoparticle tracking analysis (NTA), MSC-EVs had a particle concentration of 1.61 × 10^11^ ± 1.37 × 10^10^ particles/mL, with a mean particle diameter of 113.9 ± 9.5 nm and a mode size of 80.9 ± 33.2 nm (Figure 1A). NK-EVs exhibited a particle concentration of 1.68 × 10^11^ ± 1.11 × 10^10^ particles/mL, with a mean particle diameter of 125.2 ± 1.2 nm and a mode size of 120.1 ± 4.8 nm (Figure 1B). Both MSC-EVs and NK-EVs displayed particle size distributions predominantly within the expected extracellular vesicle size range, with the majority of particles measuring approximately 50–200 nm in diameter.

Scanning electron microscopy (SEM) further confirmed the vesicular morphology of both MSC-EVs and NK-EVs. The particles exhibited the characteristic round, membrane-bound appearance of extracellular vesicles and were observed within the size range detected by nanoparticle tracking analysis (NTA). Minor differences between SEM- and NTA-derived size measurements are expected, as NTA measures the hydrodynamic diameter of particles in suspension, whereas SEM visualizes the physical morphology and size of dehydrated vesicles (Figure 1C,D).

In western blot analysis, EV-associated markers, including apoptosis-linked gene 2–interacting protein X (ALIX), cluster of differentiation 63 (CD63) and CD81, were detected in both MSC-EVs and NK-EVs. The CD63 and CD81 panels are presented for qualitative assessment of EV marker expression and were not intended for quantitative comparison between EV preparations. However, their relative abundance varied compared with that in parental cell lysates, and consistent enrichment across all markers and EV types was not observed. The endoplasmic reticulum marker Calnexin (~90 kDa) was predominantly detected in parental cell lysates, while only weak signals were observed in EV samples (Figure 1E; Supplementary Figure S1). This pattern suggests that cellular contamination is limited but cannot be completely excluded. Collectively, these findings support the successful isolation of EVs with appropriate size distribution, morphology, and representative EV-associated protein profiles.

### 2.2. Effect of MSC-EVs and NK-EVs on Reducing Endometrial Cancer Cell Viability Dose-Dependently

To assess whether MSC-EVs and NK-EVs affect the viability of endometrial cancer cells, we exposed RL95-2 and HEC-1A cells separately to increasing concentrations of NK-EVs or MSC-EVs and evaluated their viability. The comparison between MSC-EVs and NK-EVs was not performed using matched particle concentrations. Instead, concentration ranges were selected based on preliminary dose-response experiments to identify biologically active conditions for each EV preparation.

**Figure 2:**
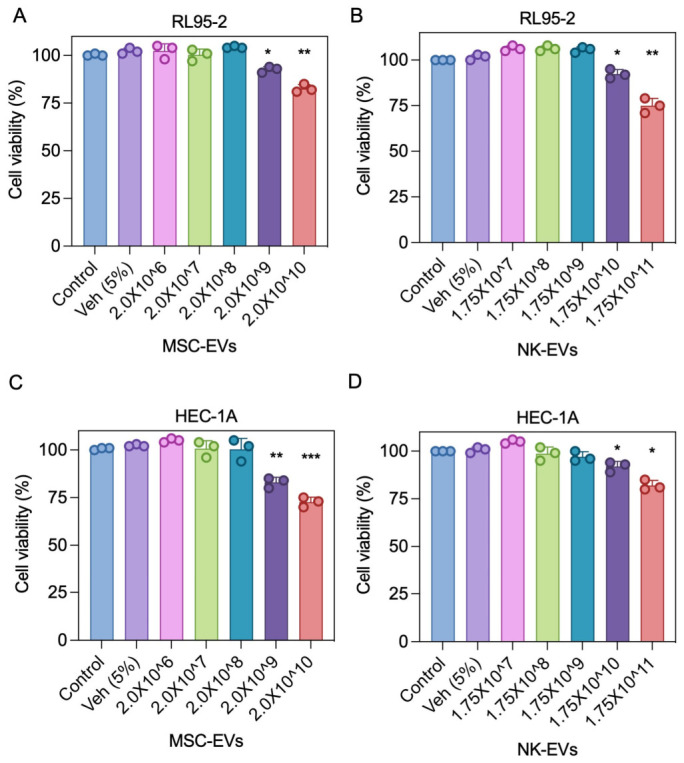
Effects of MSC-EVs and NK-EVs on endometrial cancer cell viability. (**A**,**B**) RL95-2 cells and (**C**,**D**) HEC-1A cells were treated with increasing concentrations of MSC-EVs (2.0 × 10^6^, 2.0 × 10^7^, 2.0 × 10^8^, 2.0 × 10^9^, and 2.0 × 10^10^ particles/mL) (**A**,**C**) or NK-EVs (1.75 × 10^7^, 1.75 × 10^8^, 1.75 × 10^9^, 1.75 × 10^10^, and 1.75 × 10^11^ particles/mL) (**B**,**D**), and cell viability was assessed using the CCK-8 assay. MSC-EVs reduced cell viability in a dose-dependent manner and induced significant decreases at lower particle concentrations, whereas NK-EVs produced more modest effects and required higher particle concentrations to significantly reduce cell viability. Data are presented as the mean ± standard deviation (*n* = 3). * *p* < 0.05, ** *p* < 0.01, *** *p* < 0.001 versus controls. CCK-8: Cell Counting Kit-8; MSC-EV: mesenchymal stem cell-derived extracellular vesicle; NK-EV: natural killer cell-derived extracellular vesicle. Veh: vehicle control (5% trehalose solution), used as an excipient in EV formulations to maintain vesicle stability.

In RL95-2 cells, MSC-EVs reduced cell viability in a dose-dependent manner, with significant decreases observed at 2.0 × 10^9^ and 2.0 × 10^10^ particles/mL (Figure 2A). NK-EVs exerted comparatively weaker effects at lower concentrations; however, significant reductions in cell viability were observed at 1.75 × 10^10^ and 1.75 × 10^11^ particles/mL (Figure 2B). Similarly, in HEC-1A cells, MSC-EVs significantly reduced cell viability at 2.0 × 10^9^ and 2.0 × 10^10^ particles/mL (Figure 2C). NK-EVs produced more modest effects, with significant reductions observed only at the higher tested concentrations of 1.75 × 10^10^ and 1.75 × 10^11^ particles/mL (Figure 2D). Different concentration ranges of MSC-EVs and NK-EVs were selected based on intrinsic differences in particle yield and biological activity observed in preliminary experiments. MSC-EVs exhibited measurable biological effects at lower particle concentrations, whereas NK-EVs required higher concentrations to induce reductions in cell viability.

Overall, MSC-EVs demonstrated a more pronounced reduction in cell viability compared with NK-EVs under the tested conditions, although the magnitude of these effects remained moderate.

### 2.3. Effect of MSC-EVs on Modulating Cellular Responses to Cisplatin in Cisplatin-Resistant HEC-1A Cells

**Figure 3:**
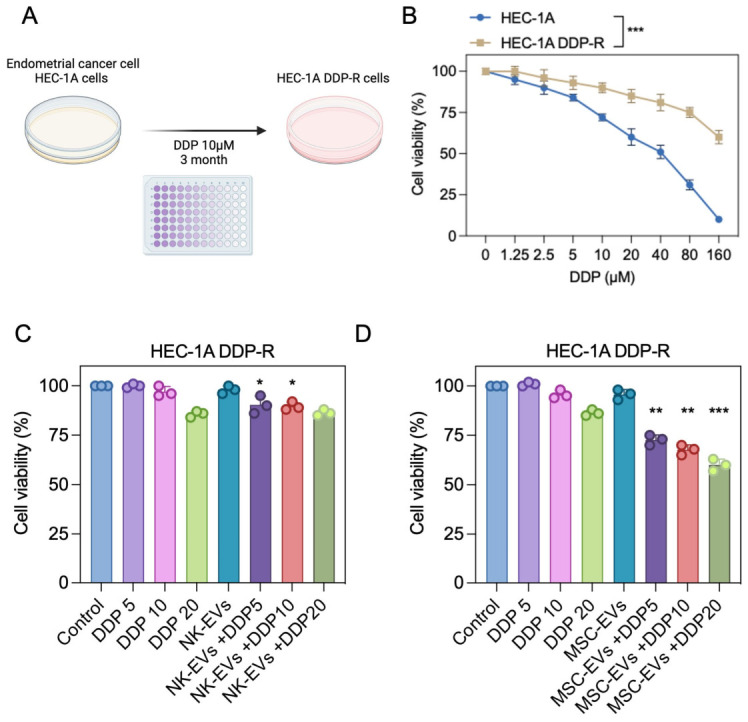
MSC-EVs modulate cellular responses to cisplatin in cisplatin-resistant HEC-1A cells. (**A**) Schematic illustration of the establishment of a cisplatin-resistant HEC-1A cell model (HEC-1A DDP-R) via continuous exposure to 10 μM cisplatin for 3 months. (**B**) Dose-response analysis showing reduced cisplatin sensitivity in HEC-1A DDP-R cells compared to parental HEC-1A cells, as assessed using the CCK-8 assay. (**C**,**D**) Effects of NK-EVs: 1.75 × 10^10^ particles/mL (**C**) and MSC-EVs: 2.0 × 10^9^ particles/mL (**D**) on cisplatin sensitivity in HEC-1A DDP-R cells. Cells were treated with MSC-EVs or NK-EVs alone or in combination with cisplatin, and their viability was measured. MSC-EVs were associated with greater reductions in cell viability under cisplatin treatment conditions, whereas NK-EVs showed comparatively modest effects. Data are presented as the mean ± standard deviation (*n* = 3). * *p* < 0.05, ** *p* < 0.01, *** *p* < 0.001. DDP: cis-diamminedichloroplatinum(II); MSC-EV: mesenchymal stem cell-derived extracellular vesicle; NK-EV: natural killer cell-derived extracellular vesicle.

To model cisplatin resistance observed in endometrial cancer, HEC-1A cells were continuously exposed to low-dose (10 μM) cisplatin for 3 months, generating a cisplatin-resistant subline, which was named ‘HEC-1A DDP-R’ (Figure 3A).

Drug-response analysis confirmed the chemoresistant phenotype of HEC-1A DDP-R. Compared to parental HEC-1A cells, HEC-1A DDP-R cells exhibited significantly higher viability across increasing cisplatin concentrations, demonstrating reduced sensitivity to the drug (Figure 3B).

Next, we examined whether EV treatment can modulate cisplatin responsiveness. Results showed that NK-EVs alone caused only modest reductions in cell viability in HEC-1A DDP-R cells. When combined with cisplatin, the cytotoxic effects of NK-EVs were slightly enhanced, but the overall impact remained limited (Figure 3C). Based on the dose-response analysis shown in Figure 2, MSC-EVs (2.0 × 10^9^ particles/mL) and NK-EVs (1.75 × 10^10^ particles/mL) were selected for subsequent cisplatin-sensitivity experiments.

In contrast, MSC-EVs were associated with greater reductions in cell viability under cisplatin treatment conditions. MSC-EV–cisplatin co-treatment led to a substantial decrease in cell viability compared with cisplatin treatment alone, with the strongest effects observed at higher cisplatin doses (Figure 3D). These findings suggest that MSC-EVs are associated with altered cellular responses to cisplatin in chemoresistant endometrial cancer cells, thereby partially reversing the chemoresistant phenotype.

### 2.4. Effect of MSC-EVs on Apoptosis- and Cell Cycle-Related Protein Expression in Cisplatin-Resistant HEC-1A Cells

**Figure 4:**
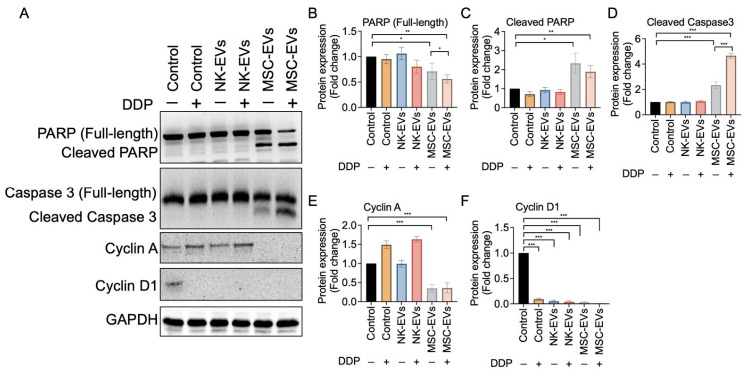
MSC-EVs promote apoptosis and suppress cell cycle progression in cisplatin-resistant HEC-1A cells. (**A**) Western blot analysis of apoptosis- and cell cycle-related proteins in HEC-1A DDP-R cells treated with NK-EVs or MSC-EVs, with or without cisplatin. Representative blots of PARP, cleaved PARP, caspase-3, cleaved caspase-3, cyclin A, cyclin D1, and GAPDH are shown. (**B**–**D**) Quantification of apoptosis markers. MSC-EVs reduced full-length PARP (**B**) and increased cleaved PARP (**C**) and cleaved caspase-3 (**D**) levels, indicating activation of apoptotic signalling. MSC-EV–cisplatin co-treatment further enhanced these effects. NK-EVs showed limited induction of apoptosis-related protein cleavage. (**E**,**F**) Quantification of cell-cycle regulators. MSC-EVs significantly decreased cyclin A (**E**) levels, while cyclin D1 (**F**) levels decreased across cisplatin, NK-EV, and MSC-EV treatments, consistent with impaired cell-cycle progression. Data are presented as the mean ± standard deviation (*n* = 3). * *p* < 0.05, ** *p* < 0.01, *** *p* < 0.001. DDP: cis-diamminedichloroplatinum(II); GAPDH: glyceraldehyde-3-phosphate dehydrogenase; MSC-EV: mesenchymal stem cell-derived extracellular vesicle; NK-EV: natural killer cell-derived extracellular vesicle; PARP: poly(ADP-ribose) polymerase.

To elucidate the mechanisms underlying EV-mediated modulation of drug sensitivity, we analysed apoptosis- and cell cycle-related protein levels in HEC-1A DDP-R cells (Figure 4A). MSC-EVs significantly reduced full-length PARP levels, with a concomitant increase in cleaved PARP levels (Figure 4B,C), indicating activation of apoptotic signalling. In addition, MSC-EVs consistently significantly elevated cleaved caspase-3 levels, further indicating induction of the caspase-dependent apoptotic pathway (Figure 4D); the combination of MSC-EVs and cisplatin further enhanced these effects. In contrast, NK-EVs exerted a minimal influence on PARP cleavage and caspase-3 activation in HEC-1A DDP-R cells, suggesting a weaker impact on apoptosis induction.

Regarding cell-cycle regulators, MSC-EVs significantly reduced cyclin A expression in HEC-1A DDP-R cells (Figure 4E), suggesting alterations in cell cycle-associated regulatory proteins. However, cisplatin, NK-EVs, and MSC-EVs all decreased cyclin D1 levels (Figure 4F), consistent with changes in proteins involved in cell-cycle regulation and reduced proliferative capacity.

**Figure 5:**
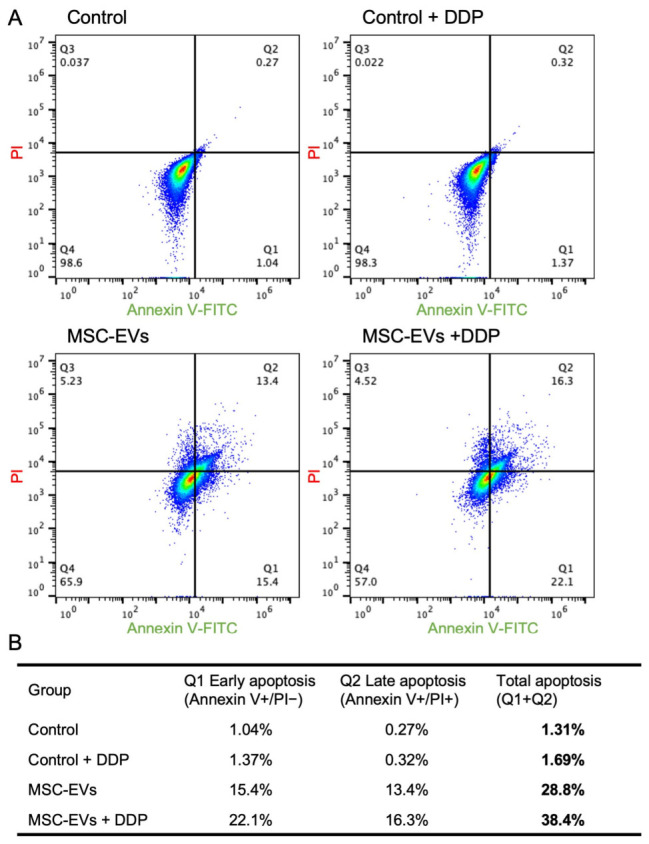
MSC-EVs are associated with increased apoptotic cell populations in cisplatin-treated chemoresistant endometrial cancer cells. HEC-1A DDP-R cells were pretreated with MSC-EVs for 24 h, followed by cisplatin (DDP; 20 μg/mL) treatment for an additional 24 h. Apoptosis was evaluated by Annexin V-FITC/PI staining followed by flow cytometric analysis. (**A**) Representative dot plots showing Annexin V-FITC (*x*-axis) and PI (*y*-axis) staining patterns under four experimental conditions: Control, Control + DDP, MSC-EVs, and MSC-EVs + DDP. Quadrants indicate viable cells (Q4, Annexin V^−^/PI^−^), early apoptotic cells (Q1, Annexin V^+^/PI^−^), late apoptotic cells (Q2, Annexin V^+^/PI^+^), and necrotic cells (Q3, Annexin V^−^/PI^+^). (**B**) Quantification of early apoptotic (Q1), late apoptotic (Q2), and total apoptotic (Q1 + Q2) cell populations. Compared with control (total apoptosis: 1.31%) and DDP-alone (1.69%) conditions, MSC-EV treatment increased the percentage of apoptotic cells (Q1 + Q2 = 28.8%), and MSC-EV + DDP co-treatment resulted in the highest proportion of apoptotic cells (Q1 + Q2 = 38.4%), consistent with apoptosis-associated molecular changes observed in western blot analyses. DDP: cis-diamminedichloroplatinum(II); MSC-EV: mesenchymal stem cell-derived extracellular vesicle; FITC: fluorescein isothiocyanate; PI: propidium iodide.

To further functionally evaluate apoptosis-related alterations observed in the western blot analysis, HEC-1A DDP-R cells were pretreated with MSC-EVs for 24 h and subsequently exposed to DDP (20 μg/mL) for an additional 24 h, followed by Annexin V-fluorescein isothiocyanate (FITC)/propidium iodide (PI) flow cytometric analysis (Figure 5A). Control and DDP-alone groups exhibited low percentages of apoptotic fractions, with total apoptotic populations (Q1+Q2) of 1.31% and 1.69%, respectively (Figure 5B). In contrast, MSC-EV treatment increased both early and late apoptotic populations, resulting in a total apoptotic fraction of 28.8%. Notably, MSC-EV + DDP co-treatment further increased the apoptotic fraction to 38.4% (Q1: 22.1%; Q2: 16.3%). These findings functionally validate the apoptosis-associated molecular changes identified by western blot analysis.

Collectively, these findings suggest that MSC-EVs are associated with reduced proliferative capacity in cisplatin-resistant endometrial cancer cells, accompanied by increased apoptotic cell populations, changes in apoptosis-related protein expression and modulation of cell cycle-related regulators. In contrast, NK-EVs predominantly influenced cell cycle-related pathways, with comparatively limited apoptosis-associated effects.

## 3. Discussion

### 3.1. EV Subtype-Specific Functional Differences

EVs derived from different cellular sources exhibit distinct biological functions depending on their molecular cargo and membrane composition. MSC-EVs have been widely studied for their immunomodulatory and tissue-regenerative properties, and emerging evidence suggests their ability to modulate tumour cell behaviour in a context-dependent manner [30,31]. MSC-EVs have influenced cell survival, proliferation, and response to chemotherapy in several cancer models, although both pro- and anti-tumour effects depending on tumour type and microenvironmental conditions have been described [23,25]. In contrast, NK-EVs, which carry cytotoxic proteins and immune effector molecules, have primarily been investigated for their immune-mediated cytotoxic potential [26,28,32,33]. However, their direct influence on the intrinsic survival pathways of tumour cells appears more limited compared to MSC-EVs. In this study, we observed that compared to NK-EVs, MSC-EVs exert stronger anti-proliferative and pro-apoptotic effects on endometrial cancer cells, highlighting that the EV subtype origin critically determines functional impact.

Cisplatin is an important chemotherapeutic for advanced or recurrent endometrial cancer; however, the development of drug resistance in the body significantly limits its long-term efficacy and contributes to treatment failure [12,34]. Mechanisms underlying cisplatin resistance include enhanced DNA repair capacity, apoptotic signalling dysregulation, altered cell-cycle control, and changes in tumour microenvironment-mediated signalling [35,36]. These adaptations allow tumour cells to evade cytotoxic stress and survive under chemotherapeutic pressure. Our cisplatin-resistant HEC-1A model highlighted this clinical challenge, demonstrating reduced drug sensitivity and sustained cell viability across increasing cisplatin concentrations. Strategies capable of re-sensitising chemoresistant tumour cells, therefore, represent a critical therapeutic need. The present study was conducted using a single cisplatin-resistant endometrial cancer cell model (HEC-1A DDP-R). Therefore, the findings should be interpreted with caution, and validation in additional endometrial cancer models will be required to determine the generalisability of the observed effects.

The distinct functional outcomes observed between MSC-EVs and NK-EVs suggest that the EV subtype origin critically influences the molecular pathways within recipient tumour cells. MSC-EVs have been reported to carry a diverse regulatory cargo, including proteins, lipids, metabolites, microRNAs, long non-coding RNAs, and signalling modulators capable of influencing intracellular stress-response pathways [30,31]. The observed increase in PARP cleavage and caspase-3 activation suggests that MSC-EVs are associated with changes in apoptotic signalling, which may contribute to increased susceptibility to cisplatin-induced cytotoxic stress. In addition, the MSC-EV-induced cyclin A and cyclin D1 reduction observed herein suggests that MSC-EVs concurrently impair cell-cycle progression, potentially limiting the capacity of chemoresistant cells to recover from DNA damage. The simultaneous activation of apoptotic signalling and proliferative driver suppression may, therefore, create an environment that favours irreversible commitment to cell death under chemotherapeutic pressure. In contrast, NK-EVs primarily reduced cyclin D1 levels, with minimal activation of apoptosis markers, indicating a predominantly cytostatic rather than a pro-apoptotic effect. This divergence may reflect differences in the EV cargo composition, as NK-EVs are often enriched in immune effector-associated molecules, whereas MSC-EVs may carry broader regulatory components affecting survival pathways. One limitation of the present study is that EV uptake efficiency was not directly evaluated. Because extracellular vesicles derived from different cellular sources may exhibit distinct internalisation kinetics, future studies employing fluorescent EV labelling and uptake analyses may help determine whether the observed biological differences are attributable to variations in EV uptake efficiency, EV cargo composition, or both.

Flow cytometry-based Annexin V/PI analysis provided additional functional validation of the apoptosis-associated molecular changes observed in this study. The concordant alterations identified across apoptotic cell populations and multiple independent protein markers support a consistent biological response rather than isolated signalling changes. Nevertheless, additional quantitative analyses, such as cell-cycle profiling or broader proteomic approaches, would further strengthen mechanistic interpretation. Taken together, these findings suggest a subtype-dependent functional specialisation of EVs and support further investigation of EV source selection in the development of EV-based strategies for chemoresistant endometrial cancer. Although Annexin V/PI analysis provided functional support for the apoptosis-associated protein changes observed following MSC-EV treatment, the experiment was performed as a targeted validation of the MSC-EV condition. Additional studies incorporating NK-EV treatment groups and quantitative statistical analyses will be required to comprehensively compare the apoptosis-related effects of different EV preparations.

The ability of MSC-EVs to modulate cellular responses to cisplatin in chemoresistant endometrial cancer cells suggests their potential value as an adjunct strategy to conventional chemotherapy. EV-based approaches may offer advantages such as biocompatibility and intrinsic targeting capabilities. However, given the context-dependent effects of MSC-EVs reported in the literature, further studies are required to define their safety profile, optimal dosing and in vivo efficacy.

Importantly, the effects of MSC-EVs in cancer are highly context-dependent. Although MSC-EVs have been reported to suppress tumour growth or enhance therapeutic responses in some experimental settings, other studies have shown that MSC-EVs can promote tumour progression, metastatic behaviour, survival signalling, angiogenesis, and drug resistance depending on their cargo composition and recipient tumour cell context. Therefore, the findings of the present study should not be interpreted as indicating a uniformly beneficial role of MSC-EVs. Instead, MSC-EVs may function as context-dependent modulators of tumour cell behaviour, and future studies should define the specific molecular cargo and biological conditions that determine whether MSC-EVs exert antitumour or protumour effects.

### 3.2. Study Limitations

This study has several limitations that should be acknowledged. First, all experiments were performed in vitro using two endometrial cancer cell lines, and only HEC-1A cells were used to establish the cisplatin-resistant model. Therefore, the generalisability of these findings to other endometrial cancer models and clinical settings requires further validation. Second, although MSC-EVs and NK-EVs exhibited differential effects on cisplatin responsiveness, the specific extracellular vesicle cargo responsible for these effects was not identified in the present study. Likewise, EV uptake/internalisation and downstream signalling pathways were not investigated, precluding definitive mechanistic conclusions. Third, the present work did not include in vivo validation; therefore, the therapeutic efficacy and safety of EV-based modulation of cisplatin responsiveness remain to be established in appropriate animal models. Finally, the current study was designed as a comparative investigation of the effects of mesenchymal stem cell-derived and natural killer cell-derived extracellular vesicles on cisplatin responsiveness rather than a comprehensive mechanistic analysis. Future studies integrating EV cargo characterisation, uptake analyses, molecular pathway validation, and in vivo models will be important for elucidating the mechanisms underlying the observed responses and for assessing their translational potential. Despite these limitations, the present study provides a comparative evaluation of MSC-EVs and NK-EVs in modulating cisplatin responsiveness in endometrial cancer cells and establishes a foundation for future mechanistic and translational investigations.

## 4. Materials and Methods

### 4.1. Cell Culture

In this study, we cultured two human endometrial cancer cell lines, RL95-2 and HEC-1A, according to standard conditions. RL95-2 cells were maintained in Dulbecco’s modified Eagle’s medium/Ham’s F-12 nutrient mixture (DMEM/F12) and HEC-1A cells in McCoy’s 5A medium, each supplemented with 10% foetal bovine serum (FBS) and 1% penicillin–streptomycin. The cells were cultured in a humidified incubator with 5% CO_2_ at 37 °C. For EV experiments, cells were cultured in a medium containing EV-depleted FBS.

### 4.2. EV Isolation

EVs derived from umbilical cord mesenchymal stem cells were isolated from conditioned medium using an automated exosome isolation system based on continuous nanofiltration technology incorporating ultrasonic-assisted purification [37,38,39]. The collection of umbilical cords and umbilical cord blood was approved by the Institutional Review Board of Tri-Service General Hospital (Taipei, Taiwan; IRB No. C202405130 and B202405124, respectively). MSCs were derived from human umbilical cord tissue and cultured under standard conditions as previously described [40,41]. NK cells were isolated from umbilical cord blood using standard negative selection/enrichment protocols, followed by expansion under defined culture conditions [42]. NK-EVs were obtained from activated NK cells cultured according to the manufacturer’s proprietary activation and expansion protocol (Precision Biotech Corp., Taipei, Taiwan). Flow cytometry characterisation of NK cells and MSCs is provided in Supplementary Figures S2 and S3.

Briefly, for large-scale exosome production, mesenchymal stem cells at passage 4 (P4) were thawed and expanded to passage 5 (P5) in CellStack-10 units. P5 cells were seeded into five CellStack-10 units at 3000 cells/cm^2^, and on Day 3, the medium was replaced with serum-free and human platelet lysate-free media for a 48 h incubation before harvest. The conditioned medium was centrifuged at 500× *g* for 10 min to remove cells and then filtered through a 0.22-μm filter to eliminate cellular debris. Next, EVs were isolated from the conditioned media of umbilical cord mesenchymal stem cells and umbilical cord blood-derived natural killer cells using the Exodus H600 system (Excellos/Aperis, San Diego, CA, USA). The entire procedure required approximately 3 h. Finally, the purified MSC-EVs were resuspended in Dulbecco’s phosphate-buffered saline and stored at −80 °C for subsequent analyses. EV preparations were thawed immediately before experiments and were not subjected to repeated freeze–thaw cycles whenever possible.

Natural killer cells were isolated from human peripheral blood mononuclear cells and then cultured under standard activation conditions. EVs were isolated from the conditioned medium collected. The supernatant underwent the same preclearing process (centrifugation at 500× *g* and filtration with a 0.22-μm filter) and automated exosome isolation for continuous nanofiltration and ultrasonic purification, as described before for MSC-EVs. This ensured comparable isolation procedures between the MSC-EVs and NK-EVs. The cell and EV preparation experiments were developed and conducted under Good Tissue Practice laboratory guidelines of the Precision Biotech Corp., Taipei, Taiwan. Although these approaches are widely used for EV characterisation, they do not directly quantify EV purity. Additional metrics, such as particle-to-protein ratios or advanced purity assessments, may provide complementary information regarding EV preparation quality.

### 4.3. Nanoparticle Tracking Analysis

NTA was performed to determine the EV size distribution and particle concentration using the NanoSight NS300 system (Malvern Panalytical, Worcestershire, UK). Briefly, the EV suspensions were diluted in particle-free phosphate-buffered saline to achieve optimal particle counts and analysed according to the manufacturer’s instructions. The mean particle size and mode diameter were recorded. Each EV preparation was analysed via NTA using three independent recordings per sample (technical replicates). In addition, NTA measurements were independently performed for each newly prepared EV sample across experiments. These measurements consistently demonstrated that the size distribution and particle concentration of the EV preparations were within the expected range for extracellular vesicles. However, it should be noted that NTA analysis provides information on particle size and concentration but does not directly assess EV purity.

### 4.4. Scanning Electron Microscopy

We examined EV morphology using SEM. Briefly, EVs were fixed with 2.5% glutaraldehyde, dehydrated through a graded ethanol series and sputter-coated with gold–palladium prior to imaging. Imaging was performed to assess EV size and shape to confirm nanoscale membrane-bound structures. Thereafter, dried samples were sputter-coated with gold–palladium and visualised using an Apreo 2S scanning electron microscope (Thermo Fisher Scientific, Waltham, MA, USA).

### 4.5. Cell Viability Assay

Briefly, RL95-2 and HEC-1A cells were separately seeded into 96-well plates and allowed to adhere overnight. Next, the cells were incubated with increasing concentrations of NK-EVs (1.75 × 10^7^–1.75 × 10^11^ particles/mL) or MSC-EVs (2 × 10^6^–2 × 10^10^ particles/mL) for 24 h (the RL95-2 and HEC-1A controls were not treated with any EV subtype). For subsequent cisplatin-sensitivity experiments, MSC-EVs and NK-EVs were used at concentrations of 2.0 × 10^9^ and 1.75 × 10^10^ particles/mL, respectively, based on the results of the initial dose-response studies. No washing step was performed following EV treatment. Subsequently, cisplatin was added at specified concentrations, and the cells were further incubated for 48 h. Next, Cell Counting Kit-8 (CCK-8) reagent was added, and the cells were again incubated for 1–2 h at 37 °C. Finally, absorbance was measured at a wavelength of 450 nm using a microplate reader. Cell viability was expressed as a percentage relative to untreated controls. All experiments were performed in triplicate (*n* = 3).

### 4.6. Establishment of a Cisplatin-Resistant Endometrial Cancer Cell Model

To establish a cisplatin-resistant endometrial cancer cell model, HEC-1A cells were continuously cultured in medium containing 10 μM cisplatin for approximately 3 months. During this period, culture medium containing cisplatin was routinely replaced, and surviving cells were maintained and expanded until a stable proliferating population was obtained. The resulting resistant population was designated as HEC-1A DDP-R. HEC-1A cells were selected for resistance induction because they exhibited stable growth and reproducible adaptation under long-term cisplatin exposure. In contrast, RL95-2 cells exhibited reduced growth stability and poor long-term survival under continuous cisplatin selection, making establishment of a stable resistant population difficult. Therefore, HEC-1A cells were chosen for subsequent resistance-model studies. Drug sensitivity was evaluated using dose-response viability assays. Briefly, parental HEC-1A and HEC-1A DDP-R cells were seeded in 96-well plates and treated with increasing concentrations of cisplatin (0–100 μM) for 48 h. Cell viability was measured using the CCK-8 assay, and dose-response curves were generated to compare sensitivity between parental and resistant cells. All experiments were performed in triplicate.

### 4.7. Western Blot Analysis

For mechanistic analysis, HEC-1A DDP-R cells were treated with NK-EVs (1.75 × 10^10^ particles/mL) and MSC-EVs (2.0 × 10^9^ particles/mL) separately for 24 h. Cells were then exposed to cisplatin (20 μg/mL) for an additional 24 h and lysed in radioimmunoprecipitation assay buffer (Thermo Fisher Scientific) containing protease inhibitors. Protein concentrations were determined using a BCA assay. Equal amounts of protein (20–30 μg) were separated on 10–12% sodium dodecyl sulphate–polyacrylamide gel electrophoresis (SDS–PAGE) gels and transferred onto polyvinylidene fluoride membranes (Millipore, Burlington, MA, USA) using a wet transfer system. Membranes were blocked with 5% non-fat milk in TBST for 1 h at room temperature and incubated overnight at 4 °C with primary antibodies against poly(ADP-ribose) polymerase (PARP; Cell Signalling Technology, Danvers, MA, USA; #9542), cleaved PARP (Cell Signalling Technology; #9542), caspase-3, cleaved caspase-3 (Cell Signalling Technology; #9661), cyclin A (Cell Signalling Technology; #67955), cyclin D1 (Cell Signalling Technology; #2922), and glyceraldehyde-3-phosphate dehydrogenase (GAPDH; Cell Signalling Technology; #2118), each diluted 1:1000. Membranes were then incubated with horseradish peroxidase (HRP)-conjugated secondary antibodies (1:5000; Santa Cruz Biotechnology, Dallas, TX, USA) for 1 h at room temperature. Signals were visualised using enhanced chemiluminescence (ECL) reagents and quantified by densitometry using ImageJ software, version 8. All experiments were performed in triplicate.

### 4.8. Annexin V/PI Staining and Flow Cytometric Analysis of Apoptosis

To functionally evaluate apoptosis, HEC-1A DDP-R cells were treated under the indicated experimental conditions. Briefly, cells were pretreated with MSC-EVs for 24 h, followed by cisplatin (DDP; 20 μg/mL) for an additional 24 h. After treatment, cells were harvested, washed twice with cold phosphate-buffered saline, and resuspended in binding buffer. Apoptosis was assessed using an Annexin V-FITC/PI Apoptosis Detection Kit (BioLegend, San Diego, CA, USA) according to the manufacturer’s instructions. Briefly, cells were incubated with Annexin V-FITC and PI staining solution for 15 min at room temperature in the dark and subsequently analysed via flow cytometry. Cell populations were classified as viable cells (Annexin V^−^/PI^−^), early apoptotic cells (Annexin V^+^/PI^−^), late apoptotic cells (Annexin V^+^/PI^+^), or necrotic cells (Annexin V^−^/PI^+^). Data were analysed using FlowJo software (version 10.8.1; FlowJo LLC, Ashland, OR, USA). All experiments were independently performed in triplicate.

### 4.9. Statistical Analysis

Data are presented as the mean ± standard deviation (SD). Statistical analyses were performed using GraphPad Prism software (version 10; GraphPad Software, San Diego, CA, USA). Comparisons between two groups were analysed using Student’s *t*-test. Comparisons among multiple groups were analysed using one-way analysis of variance (ANOVA) followed by Tukey’s multiple-comparisons test. A *p*-value < 0.05 was considered statistically significant. All experiments were independently performed at least three times (*n* = 3).

## 5. Conclusions

In summary, MSC-EVs and NK-EVs exhibit subtype-specific biological activities in endometrial cancer cells. MSC-EVs were associated with greater reductions in cell viability, increased apoptotic cell populations, and coordinated changes in apoptosis- and cell cycle-related markers compared with NK-EVs, whereas NK-EVs predominantly influenced cell cycle-related proteins with comparatively limited apoptosis-associated effects. These findings highlight the importance of EV subtype origin in determining functional outcomes and support further investigation of MSC-EVs as a potential EV-based approach for modulating cellular responses in chemoresistant endometrial cancer.

## Data Availability

The original contributions presented in this study are included in the article and its Supplementary Materials. Further inquiries can be directed to the corresponding author.

## Supplementary Materials

The following supporting information can be downloaded at: https://www.mdpi.com/article/10.3390/ijms27135842/s1.

## Abbreviations

The following abbreviations are used in this manuscript:

EV	Extracellular vesicles
FBS	FoEvetal bovine serum
NTA	Nanoparticle tracking analysis
SD	Standard deviation
SEM	Scanning electron microscopy
