# Supplementary material for: Differential Effects of Mesenchymal Stem Cell- and Natural Killer Cell-Derived Extracellular Vesicles on Cisplatin Responsiveness in Endometrial Cancer Cells
Source: Int J Mol Sci. 2026 Jun 28;27(13):5842. doi: 10.3390/ijms27135842 (PMC13361685; doi:10.3390/ijms27135842)
Supplement: Supplementary file 1 [file ijms-27-05842-s001.zip › ijms-4378413-Supplementary.pdf]

Supplementary Figure S1

Fig. 4A

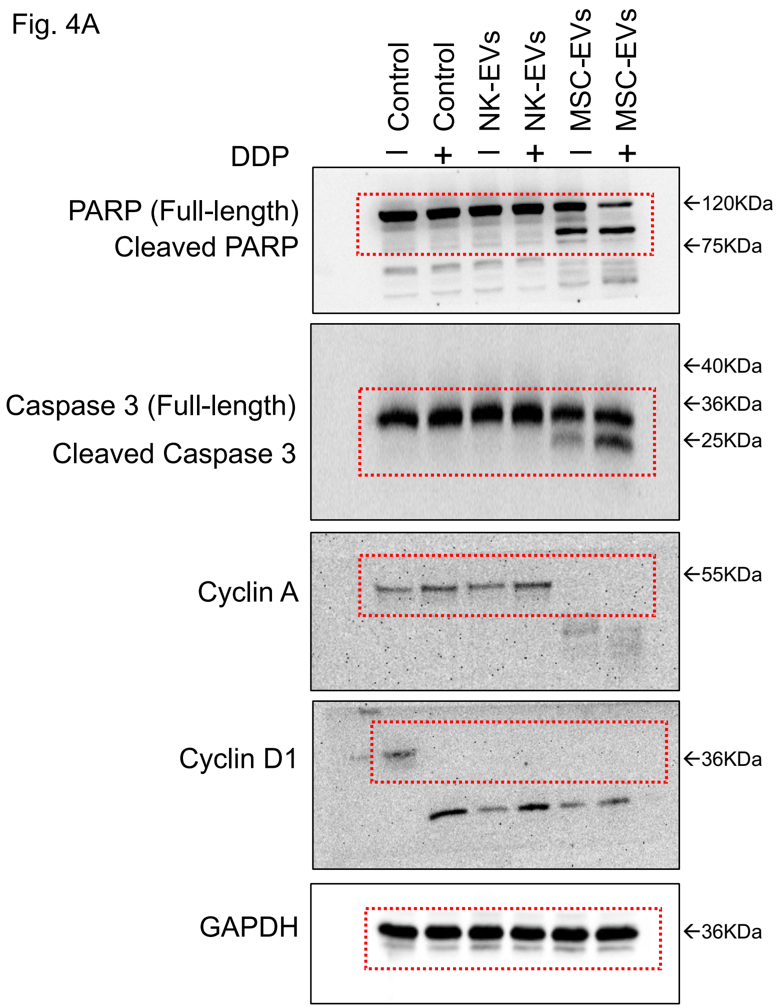

Fig. 1E

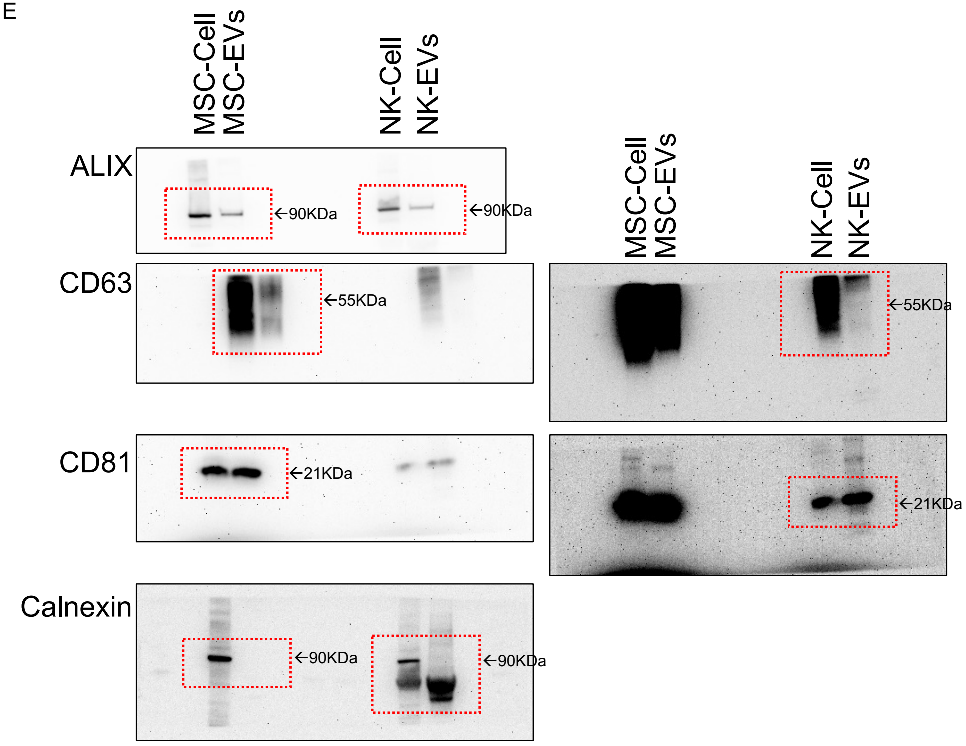

Supplementary Figures S2

TSIRB2024_TS02-003-UcBNK-20241105-Ct-F20W-D29
(Frozen 20 weeks, 3_PB_0,5xC1C2_beads, PB101 with RBC Lysis, 6 color)

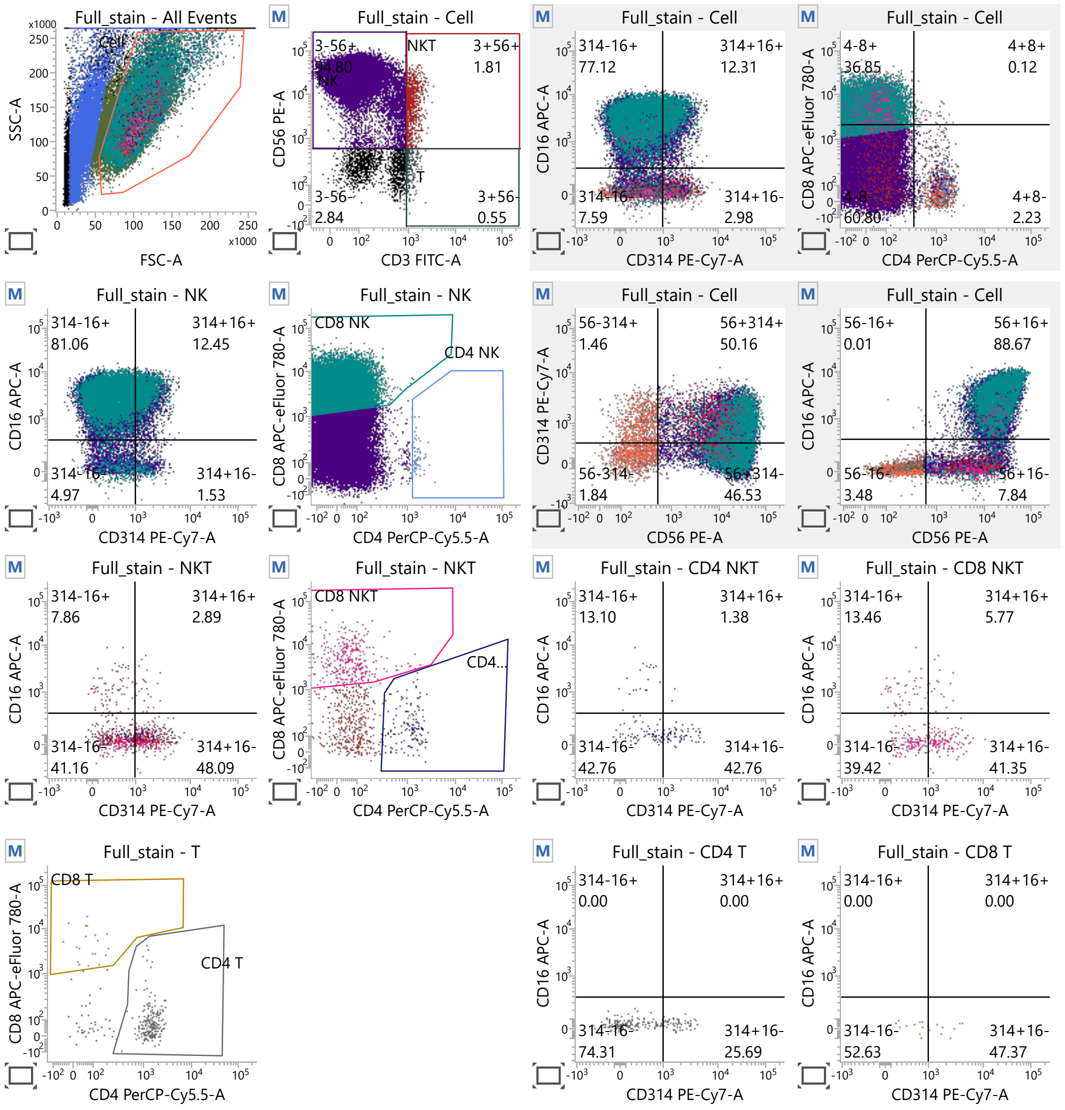

Supplementary Figures S3

PQC date: 4/24/2025 11:26:52 AM
CS&T bead lot: 3277700
CS&T bead expiration date: 10/31/2025 12:00:00 AM
PQC status: Pass

Acquisition date: 4/24/2025 11:46:24 AM
Tube Settings: 20240425_6C

MNC = 62.2%
Pecentage to MNC:
NK = 94.8%
NKT = 1.8%
T= 0.5%

CD314+ CD16+ NK = 11.8%

Percentage to parent:
CD4+ NKT = 16.8%
CD8+ NKT = 36.1%

CD4+ T = 79.6%
CD8+ T = 6.9%

CD8+ NK = 49.3%

Negative controls

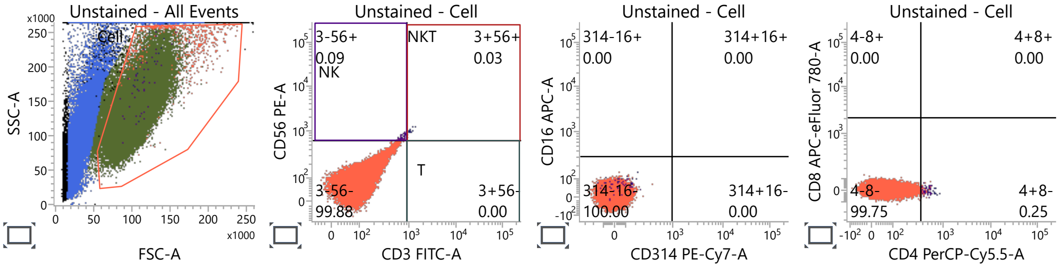

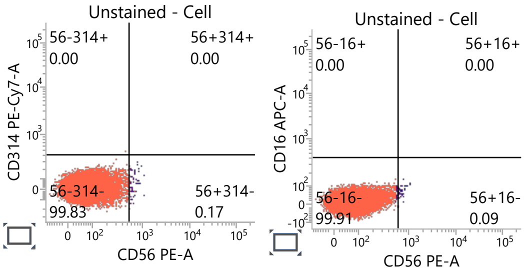

Statistics

Name	Events	% Parent	% Grandparent	% Total
Unstained:All Events	81,388	***	***	100.00
Unstained:Cell	50,000	61.43	***	61.43
